# The Effect of a Sustained High-Fat Diet on the Metabolism of White and Brown Adipose Tissue and Its Impact on Insulin Resistance: A Selected Time Point Cross-Sectional Study

**DOI:** 10.3390/ijms222413639

**Published:** 2021-12-20

**Authors:** Babu Raja Maharjan, Susan V. McLennan, Christine Yee, Stephen M. Twigg, Paul F. Williams

**Affiliations:** 1 Greg Brown Diabetes & Endocrinology Laboratory, Sydney Medical School, University of Sydney, Sydney, NSW 2006, Australia; susan.mclennan@health.nsw.gov.au (S.V.M.); cyeephd@gmail.com (C.Y.); stephen.twigg@sydney.edu.au (S.M.T.); 2 Department of Biochemistry, School of Medicine, Patan Academy of Health Sciences, Lalitpur 44700, Nepal; 3 New South Wales Health Pathology, Sydney, NSW 2050, Australia; 4 Department of Endocrinology, Royal Prince Alfred Hospital, Sydney, NSW 2006, Australia

**Keywords:** long high-fat diet, fat cell dysfunctionality, ectopic lipid deposition, insulin resistance

## Abstract

(1) Background: studies on the long-term dynamic changes in fat depot metabolism in response to a high-fat diet (HFD) on hepatic lipid deposition and insulin resistance are sparse. This study investigated the dynamic changes produced by HFD and the production of dysfunctional fat depots on insulin resistance and liver lipid metabolism. (2) Methods: mice fed a chow or HFD (45% kcal fat) diet had three fat depots, liver, and blood collected at 6, 10, 20, and 30 weeks. Anthropometric changes and gene markers for adipogenesis, thermogenesis, ECM remodeling, inflammation, and tissue insulin resistance were measured. (3) Results: early responses to the HFD were increased body weight, minor deposition of lipid in liver, increased adipocyte size, and adipogenesis. Later changes were dysfunctional adipose depots, increased liver fat, insulin resistance (shown by changes in ITT) accompanied by increased inflammatory markers, increased fibrosis (fibrosis > 2-fold, *p* < 0.05 from week 6), and the presence of crown cells in white fat depots. Later, changes did not increase thermogenic markers in response to the increased calories and decreased UCP1 and PRDM16 proteins in WAT. (4) Conclusions: HFD feeding initially increased adipocyte diameter and number, but later changes caused adipose depots to become dysfunctional, restricting adipose tissue expansion, changing the brown/beige ratios in adipose depots, and causing ectopic lipid deposition and insulin resistance.

## 1. Introduction

During times of nutrient scarcity, having sufficient storage capacity to cope with changes in dietary calorie intake is important [1]. In a normal healthy condition, adipose tissue exhibits remarkable plasticity and dynamically responds to accumulate or to release lipid stores, depending on nutrient availability and hormonal signaling [2]. However, current dietary habits and the availability of cheap and calorie-dense food in modern society has disrupted this balance [1,3] and led to an alarming increase in the prevalence of obesity type 2 diabetes and insulin resistance worldwide [4]. The increase in fat deposition in ectopic tissues can result in tissue inflammation, insulin resistance, and ultimately, systemic metabolic dysfunction [2], but as we and others have found, there are many unanswered questions linking the dynamics and time course of adipose tissue dysfunction with ectopic lipid deposition and the advent of impaired insulin action [5,6].

There is a growing trend to develop therapeutic strategies to induce the “browning” of WAT in humans as a treatment for obesity [7]. Short-term studies have established the role of inappropriate ectopic fat deposition with the onset of insulin resistance [8]. However, it remains unclear if therapy that increased beiging of fat would be effective in combating the long-term effects of HFD (>14 weeks), as studies on mitochondrial biogenesis and thermogenesis in adipose tissue have been inconclusive. While several studies have demonstrated that a HFD diet increases thermogenic activity through sympathetic stimulation in brown adipose tissue (BAT) [9], others have demonstrated suppressed adrenergic signaling with downregulated mitochondrial biogenesis in a single fat cell or in white and brown adipose tissue depots [10]. Clarification as to whether divergent results were due to: (1) differences in the choice or in the diversity and number of fat depots studied; (2) differences in time course; or (3) differences in the responses of selected white adipose tissue depots is clearly needed.

Traditionally, chronic inflammation in adipose tissue has been associated with fibrotic changes [11] and the development of insulin resistance [12]. More recently, a role of acute inflammation to allow adipose tissue remodeling and expansion has been elucidated [13]. It is unclear at what point in obesity the inflammation initiated adipose tissue expansion or increased fibrosis, because studies concentrated on single fat cell depots and did not determine if similar changes were occurring in all fat depots. This extended cross-sectional study at different time points of HFD feeding examined the dynamic changes in fat cell depots over a 30-week HFD, to determine if there was diversity/similarity in the metabolic responses of individual fat depots, and if these changes had effects on other tissues or on whole-body metabolism in response to the HFD compared with age-matched chow-fed mice.

The “lipid spillover” hypothesis predicts that excess fat storage becomes prominent in ectopic sites (viscera, heart, and vasculature) that have exceeded the limits in the storage capacity of adipose tissue depots, thus contributing to insulin resistance [14,15]. Studies of ECM remodeling showed increased collagen VI in the adipose tissue of obese individuals led to fibrosis that restricted adipose tissue expansion, resulting in ectopic fat deposition and insulin resistance [16]. However, as described earlier, fibrosis and inflammation are essential for adipose tissue expansion, so the critical timing of the functional changes that decrease the capacity of SAT, WAT, and BAT to handle lipids safely and allow lipid overflow to cause ectopic fat deposition in preclinical models has yet to be clarified.

Although obesity is associated with a greater risk of mortality [17], the development of the metabolically healthy obesity concept [18] and of a protective role of subcutaneous fat [19] has seen the development of different concepts to address the heterogeneous health outcomes seen in the obese. Each individual fat depot is a complex mixture of cells that contribute to the functional heterogeneity observed within and between the fat depots in different anatomical locations [15]. However, the studies have usually been in a single depot and at a limited number of time points in the response to the HFD. Longer-term studies coordinating the temporal changes of the metabolic responses of fat depots viz. (namely) adipogenesis, thermogenesis, inflammation, and fibrosis in response to HFD and the development of insulin resistance are rare; however, such studies are essential to understand the dynamic interactions and mechanisms that underlie the progressive metabolic dysfunction caused by a HFD. In particular, we need to understand why the fat depots initially manage the storage of excess lipid and how the progressive loss of this function can lead to a metabolic imbalance and to the development of insulin resistance in a manner similar to the fat cell GLUT4 knockout [5]. Our study was a cross-sectional study at different time points of HFD feeding (ad libitum) over 30 weeks on the functional changes in SAT, EPI, and BAT and the consequence of their dysfunctionality on liver and the metabolic phenotype.

## 2. Results

### 2.1. Anthropometric Measurements

**Figure 1:**
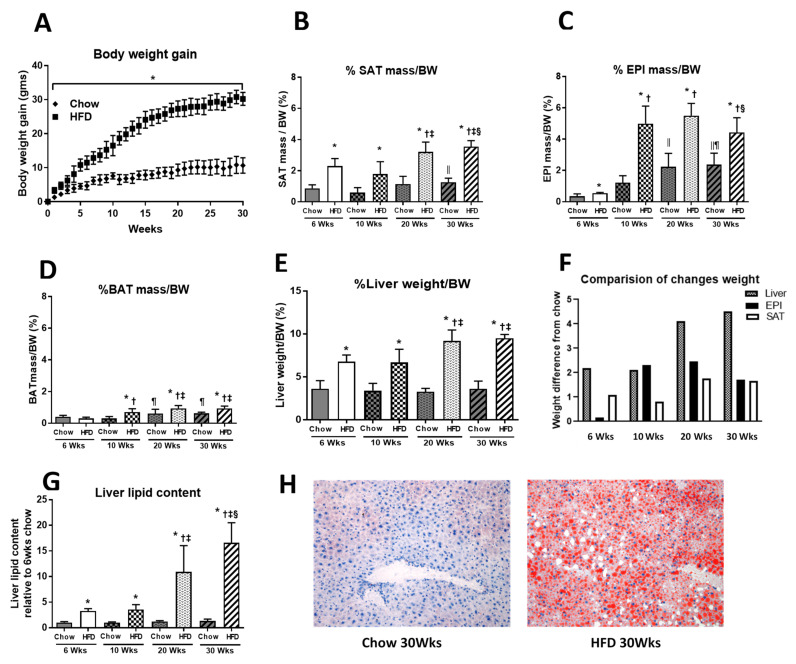
(**A**) Body weight gain in Chow and HFD groups in each week from the beginning of HFD feeding to 30 weeks; (**B**) SAT mass; (**C**) EPI mass; (**D**) BAT mass; (**E**) liver weight expressed in a percentage of body weight (BW); (**G**) lipid content in the liver; (**H**) lipid content in the liver at 30 weeks of HFD feeding (pictures taken at 200× magnification). Data are expressed as mean ± SD. An unpaired *t*-test was used to compare Chow and HFD at each time point, and one-way ANOVA with Tukey’s multiple comparison test was used to compare within the Chow or HFD groups across different time points. *p*-value < 0.05 * vs. respective Chow, ǁ vs. 6 weeks Chow, ¶ vs. 10 weeks Chow, † vs. HFD 6 weeks, ‡ vs. HFD 10 weeks, § vs. HFD 20 weeks. (**F**) Data expressed as mean difference of chow and HFD at respective time points for liver, EPI, and SAT.

The body weight of chow-fed mice increased progressively from 22.04 ± 1.6 g to 33.5 ± 1.9 g over 30 weeks, gaining on average 0.38 g/week over 1–6 weeks, increasing to 0.95 g/week and decreasing in the last few weeks to 0.24 g/week. The body weight of HFD-fed mice increased from 22.72 ± 1.4 to 52.4 ± 2.2 g over the 30 weeks, with an average initial increase in weight of 0.90 g/week, increasing significantly up to week 15 to 1.67 g/week, but then decreasing to 0.60 g/week from 15 to 30 weeks (Figure 1A). In chow-fed mice, whole-body weight was significantly increased at week 20 (34 ± 1.9 g) and week 30 (34 ± 1.8 g) compared to that in week 6 (28 ± 0.6 g) and week 10 (29 ± 1.3 g) (*p* < 0.05). Similarly in HFD-fed mice, whole-body weight was significantly increased at week 20 (48 ± 5.0 g) compared to that in week 6 (36 ± 2.3 g) and week 10 (39 ± 4.2 g), and a further increase was observed at week 30 (52 ± 2.2 g) (*p* < 0.05) (Figure S1).

The increased body weight in response to HFD was accompanied by an increase in mass of all three of the fat depots (Figure 1B–D). However, SAT mass increased earlier by week 6 (HFD: 0.89 ± 0.21 g, Chow 0.2 ± 0.03 g), and remained constant until 10 weeks. EPI mass increased from 0.20 ± 0.03 to 1.95 ± 0.5 g in HFD compared to Chow, which had minor increases from 0.10 ± 0.04 to 0.36 ± 0.13 g. From 10–20 weeks, SAT increased to 1.64 ± 0.37 g, but tapered to 1.80 ± 0.27 g at 30 weeks in HFD. A pronounced increase in EPI occurred to week 20 (2.65 ± 0.44 g), but after this, EPI weight tapered to 2.31 ± 0.43 g at week 30 (Figure 1C). Only small changes in mass were seen in BAT, but there was a significant change at 10 weeks compared to Chow, which plateaued at weeks 20 to 30 (Figure 1D). In chow-fed animals, there were small increases in SAT and BAT mass over 30 weeks, but EPI demonstrated a progressive increase from 0.5 to ~2% of body mass at 20 weeks, plateauing to 30 weeks (Figure 1B–D).

Liver weight also increased significantly at 6, 10, 20, and 30 weeks of HFD, reaching ~6% of body weight at 6 weeks compared to 3% in Chow (Figure 1E). This was maintained from week 6 to week 10, and it reached 9% of body weight by week 20. The increase in liver weight was accompanied by increased lipid content (Figure 1G,H), which was 3-fold greater at week 10, reaching 10-fold at 20 weeks of HFD, and further increasing to 12-fold at 30 weeks, when compared to Chow. The increased liver weight and lipid content at 20 weeks coincided with the plateau and decline in fat pad weights seen between 20 and 30 weeks (Figure 1B,C).

However, a better perspective of the dynamics of the changes that were occurring in response to the HFD were seen when we considered these changes against the same changes in chow-fed mice. By subtracting chow-fed changes from HFD, we defined the dynamics of these changes during this study (Figure 1F). At 6 weeks, EPI had not increased compared to chow, but SAT had increased by 1% BW over Chow. At 10 weeks, liver and SAT had minimal changes, but EPI increased by over 2% BW over Chow. At 20 weeks, EPI was unchanged, but liver and SAT increased by 4% BW and 2% BW, respectively. Compared to 20 weeks, at 30 weeks a decline in fat pad weight was seen in EPI and SAT, where an increase of 0.5 % BW occurred in liver, with a marked increase in lipid in that tissue seen with the changes in ITT (Figure 1G,H).

**Figure 2:**
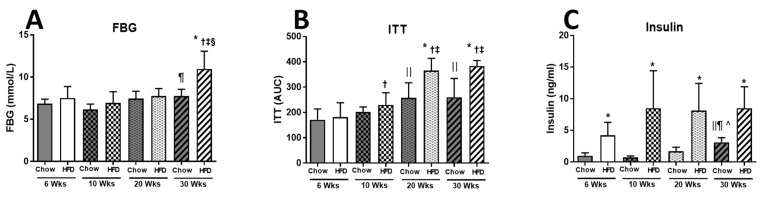
(**A**) Fasting blood glucose (FBG); (**B**) insulin tolerance test (ITT); (**C**) plasma insulin level in Chow and HFD at 6, 10, 20, and 30 weeks of HFD feeding. Data are expressed as mean ± SD. An unpaired *t*-test was used to compare Chow and HFD of each time point, and one-way ANOVA with Tukey’s multiple comparison test was used to compare within Chow or HFD groups across different time points. *p*-value < 0.05 * vs. respective Chow, ǁ vs. 6 weeks Chow, ¶ vs. 10 weeks Chow, ^ Chow 20 weeks, † vs. HFD 6 weeks, ‡ vs. HFD 10 weeks, § vs. HFD 20 weeks.

It was at this crucial time of 20 to 30 weeks when we related these changes in liver to the changes in fat cell diameter and number, and to whole-body insulin resistance seen in the changes in ITT. The early changes in weight were associated with increased plasma insulin levels at 6 weeks of HFD (Chow 1.00 ± 0.44 and HFD 4.23 ± 2.04 ng/mL), which increased until week 10, but then plateaued. The fasting blood glucose was maintained at near normal levels without further increases in insulin to week 20, but at week 30, blood glucose had risen (Figure 2C). At this time, liver had increased in mass and lipid content, fat pads had declined in weight (20–30 weeks), and the area under the ITT curve (AUC, indicating resistance to insulin action) had increased compared to chow-fed mice (Figure 1F–H and Figure 2B). There was a minor increase in the ITT (AUC) at week 10 in HFD that became significant at 20 weeks, reaching 260 ± 74 a.u. in Chow vs. 383 ± 22 a.u. in HFD. Despite an increase in ITT being present in both Chow and HFD mice, it became pronounced from 20 weeks of HFD, and FBG became significantly elevated at 30 weeks of HFD (Chow 7.74 ± 0.82 vs. HFD 10.96 ± 2.12 mmol/L) (Figure 2A–C).

**Figure 3:**
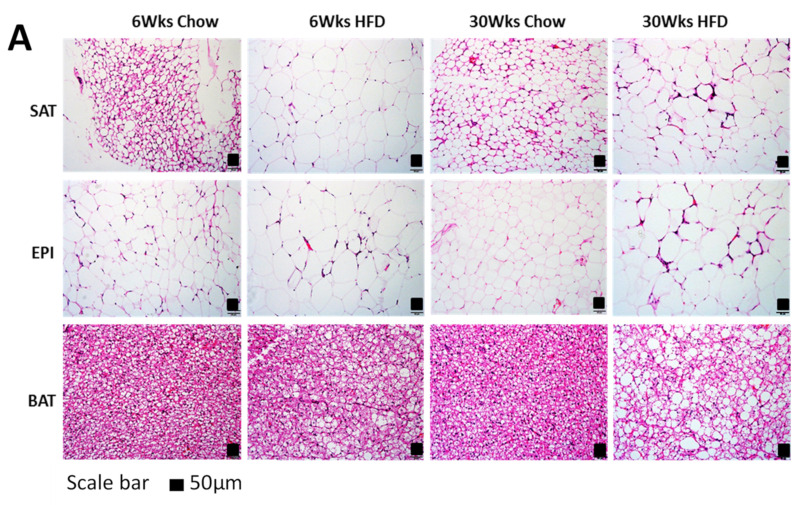

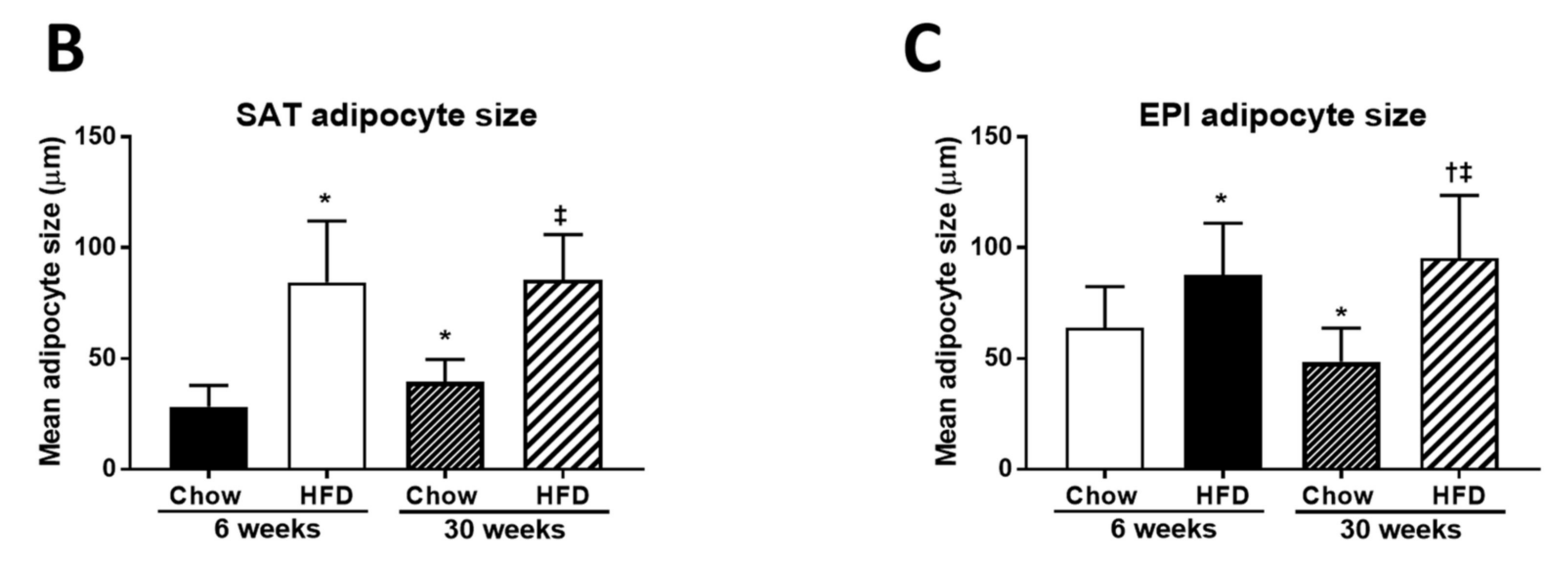
(**A**) H&E staining of paraffin section of fat tissues (SAT, EPI, and BAT). Mean adipocyte size: (**B**) SAT; (**C**) EPI of Chow and HFD at 6 and 30 weeks of HFD feeding. Data are expressed as mean ± SD. Two-way ANOVA with Sidak’s multiple comparison test was used. *p*-value < 0.05 * vs. 6 weeks Chow, † vs. 6 weeks HFD, ‡ vs. 30 weeks Chow.

Early in the progressive changes in the fat mass of both the SAT and EPI from 6 to 30 weeks HFD, they were accompanied by increases in the adipocyte size, indicating hypertrophy compared to Chow. The increase in SAT fat cell size is shown in Figure 3A,B: the cell diameter at week 6 was 28 ± 10 µm in Chow and was 84 ± 28 µm in HFD. After this initial increase, there was only a further small increment to 88 ± 21 µm at week 30 of the HFD, indicating a diminished capacity to increase their size, because Chow cell diameter, although smaller, continually increased to 40 ± 10 µm. A similar pattern was seen for EPI, with Chow cell diameter being smaller, at 64 ± 19 µm at week 6 compared to 88 ± 23 µm in HFD (Figure 3A,C). In contrast to SAT, EPI in chow underwent a decrease to 49 ± 15 µm by week 30, but HFD mice showed a small increase from 88 µm to 95 ± 28 µm. A decrease in EPI cell diameter coupled with increased mass in the Chow mice reflected the ability of this fat cell depot to increase in number, whereas the HFD animals had an early increase in size and mass of the depots that became limited in the latter stages of the study. Changes in BAT over the whole course of the study were unremarkable.

### 2.2. Adipogenesis Markers

**Figure 4:**
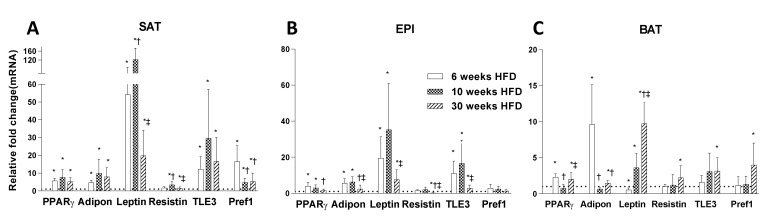
Comparison of expression of genes regulating adipogenesis (*Pparg*, adiponectin, leptin, resistin, *Tle3*, and *Pref1*) in SAT (**A**), EPI (**B**), and BAT (**C**) at 6, 10, and 30 weeks of HFD feeding. Data expressed as mean ± SD relative to the respective Chow at each time point. An unpaired *t*-test was used to compare between Chow and HFD at each time point, and one-way ANOVA with Tukey’s multiple comparison test was used to compare among the HFD cohort of three different time points. *p*-value < 0.05 * vs. respective Chow, † vs. HFD 6 weeks, ‡ vs. HFD 10 weeks.

Consistent with the HFD-induced changes of fat pad mass and adipocyte size in SAT, EPI, and BAT, there were pronounced initial increases in the expression of genes regulating adipogenesis (*Pparg*, *Tle3*, adiponectin, leptin, resistin), peaking in all three fat depots compared to chow at 10 weeks HFD, but decreasing significantly from 10 to 30 weeks (Figure 4A–C). There were distinct increases in the expression of genes regulating adipogenesis in SAT and EPI from 6 and 10 weeks, but the change was significantly greater in SAT than in EPI or in BAT. Leptin and *Tle3* underwent a 2-fold increase, and *Pref1* a 17-fold increase in SAT, with a smaller increase in BAT that was not seen in EPI. The decreased response in the expression of genes regulating adipogenesis at the latter stages of the study indicated the presence of dysfunctionality in the white fat pads. This was related to the absence of an increase in adipokines despite an increase in adipocyte size and number. In BAT, there was a modest effect of HFD on expression of genes regulating adipogenesis (with the exception of adiponectin and leptin), which showed a progressive increase over the whole cross-sectional study. Interestingly, the preadipocyte marker *Pref1* was also increased in BAT and SAT, but was minimal in EPI (Figure 4A–C).

### 2.3. Thermogenesis Markers

**Figure 5:**
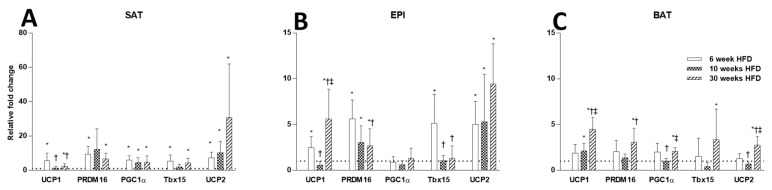
Comparison of thermogenic markers (*Ucp1*, *Prdm16*, *Pgc1a*, *Tbx15*, *and Ucp2*) at 6, 10, and 30 weeks of HFD feeding in SAT (**A**), EPI (**B**), and BAT (**C**). Data expressed as mean ± SD relative to the respective Chow at each time point. AN unpaired *t*-test was used to compare between Chow and HFD at each time point, and one-way ANOVA with Tukey’s multiple comparison test was used to compare among the HFD cohort of three different time points. *p*-value < 0.05 * vs. respective Chow, † vs. HFD 6 weeks, ‡ vs. HFD 10 weeks.

**Figure 6:**
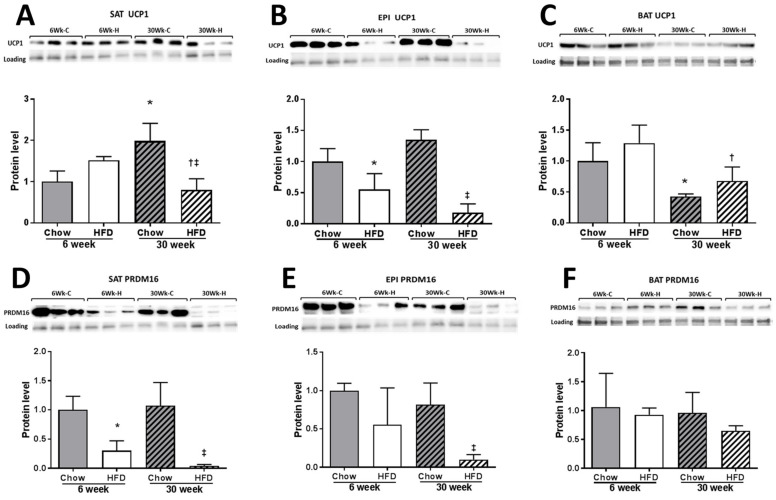
Western blot of UCP1 protein in SAT (**A**), EPI (**B**), and BAT (**C**); and PRDM16 protein in SAT (**D**), EPI (**E**), and BAT (**F**) at 6 weeks and 30 weeks of HFD with chow-fed control. Data expressed as mean ± SD relative to 6 weeks Chow. Two-way ANOVA with Sidak’s multiple comparison test was used. *p*-value < 0.05 * vs. 6 weeks Chow, † vs. 6 weeks HFD, ‡ vs. 30 weeks Chow. A UCP1 protein band was detected at 33 kDa, and PRDM16 protein at 140 kDa.

While the HFD increased the mRNA levels for the thermogenic markers in all three fat depots, the time course and the magnitude of these changes in the three different fat cell depots was not similar (Figure 5A–C). In SAT, the HFD produced a marked increase in the mRNA level of the thermogenic markers at week 6 and compared to Chow (note different scale in SAT to EPI and BAT), and they all remained elevated until week 30. In EPI, smaller increases in the mRNA levels of thermogenic markers were observed and maintained over the 30 weeks. The exception in EPI was the *Pgc1a* mRNA, which remained unchanged by the HFD. In BAT, *Ucp1* mRNA increased at week 10 of HFD feeding and continued to increase to week 30, when it reached comparable increases to EPI. At week 30, in BAT a consistent increase in *Ucp1* mRNA was accompanied by a rise in the mRNA of its regulators *Prdm16*, *Pgc1a*, *and Tbx15*. In an unexpected result, the increased *Ucp1* mRNA was not accompanied by an increase in UCP1 protein in any of the three fat depots. In SAT, UCP1 protein was comparable to Chow at week 6, but along with the PRDM16 protein (Figure 6A,D), had decreased significantly by week 30. In EPI, the UCP1 and PRDM16 proteins were reduced at all time points of HFD (Figure 6B,E). In BAT, changes in UCP1 and PRDM16 protein levels were not seen at any of the time points (Figure 6C,F). Interestingly, *Ucp2* mRNA steadily increased over time in all the fat depots (Figure 5A–C).

### 2.4. ECM Remodeling Markers

**Figure 7:**
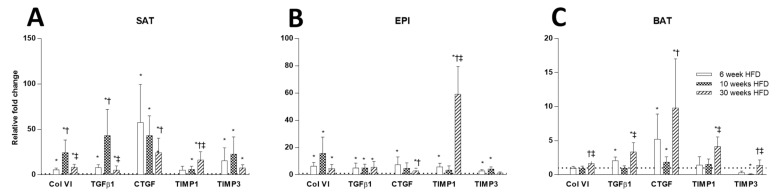
Comparison of ECM remodeling markers (collagen VI, *Tgfb1*, *Ccn2/Ctgf*, *Timp1*, and *Timp3*) in SAT (**A**), EPI (**B**), and BAT (**C**) at 6, 10, and 30 weeks of HFD feeding. Data expressed as mean ± SD relative to the respective Chow at each time point. An unpaired *t*-test was used to compare between Chow and HFD at each time point, and one-way ANOVA with Tukey’s multiple comparison test was used to compare among the HFD cohort of three different time points. *p*-value < 0.05 * vs. respective Chow, † vs. HFD 6 weeks, ‡ vs. HFD 10 weeks.

**Figure 8:**
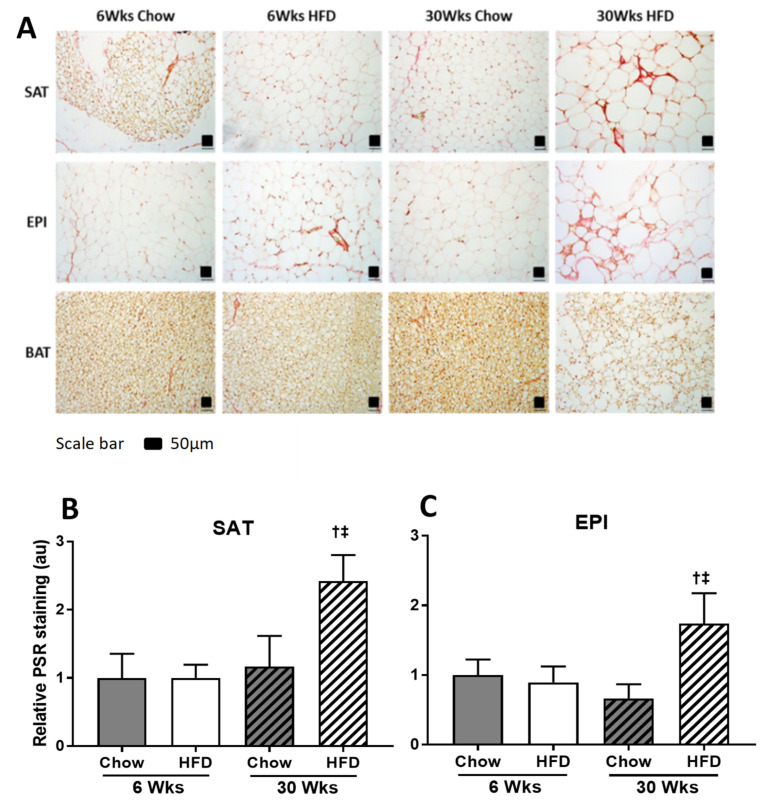
(**A**) PSR staining of SAT, EPI, and BAT at 6 weeks and 30 weeks of HFD with chow-fed control. PSR staining quantification of SAT (**B**) and EPI (**C**). Data expressed as mean ± SD relative to 6 weeks Chow. Two-way ANOVA with Sidak’s multiple comparison test was used. *p*-value < 0.05 vs. 6 weeks Chow, † vs. 6 weeks HFD, ‡ vs. 30 weeks Chow. au: arbitrary unit.

**Figure 9:**
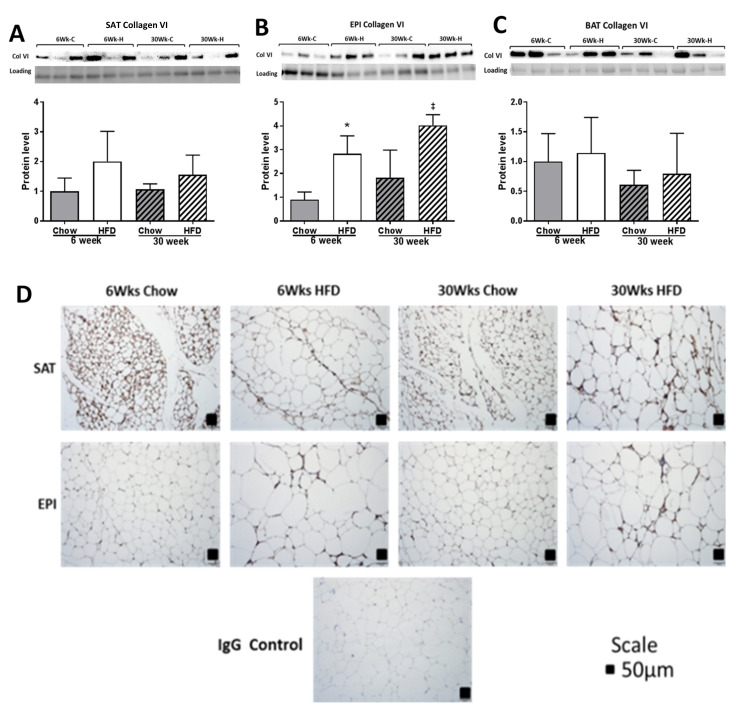
Western blot of collagen VI in SAT (**A**), EPI (**B**), and BAT (**C**); IHC staining for collagen VI (**D**) in SAT and EPI at 6 weeks and 30 weeks of HFD with chow-fed control. Data expressed as mean ± SD relative to 6 weeks Chow. Two-way ANOVA with Sidak’s multiple comparison test was used. *p*-value < 0.05 * vs. 6 weeks Chow, ‡ vs. 30 weeks Chow. A collagen VI protein band was detected at 140 kDa.

The effect of HFD on remodeling ECM markers was significant, and was greater in EPI than in SAT, and with only smaller effects observed in BAT. Significant fibrotic changes were observed in SAT and EPI at week 30 of HFD, as shown by the increased collagen VI mRNA (Figure 7A,B), PSR staining (Figure 8A–C), collagen VI protein (Figure 9A–D), and increased mRNA of the fibrotic regulators *Tgfb1*, *Ccn2/Ctgf*, *Timp1*, and *Timp3* (Figure 7A,B). In EPI, changes were seen early at week 6 of the HFD, when collagen VI mRNA and protein were increased significantly, along with the mRNA levels of the fibrotic regulators *Tgfb1*, *Ccn2/Ctgf*, *Timp1*, and *Timp3*. In contrast, in SAT, although there were significant increases in mRNA for collagen VI and in the fibrotic regulators, collagen VI protein did not change. However, in BAT, while the HFD did not show a significant change in collagen VI mRNA (Figure 7C) or in collagen protein (Figure 9C), the mRNA of the fibrotic and differentiation regulators *Tgfb1* and *Ccn2/Ctgf* were increased at both 6 and 30 weeks, together with increased mRNA for *Timp1* (Figure 7C).

**Figure 10:**
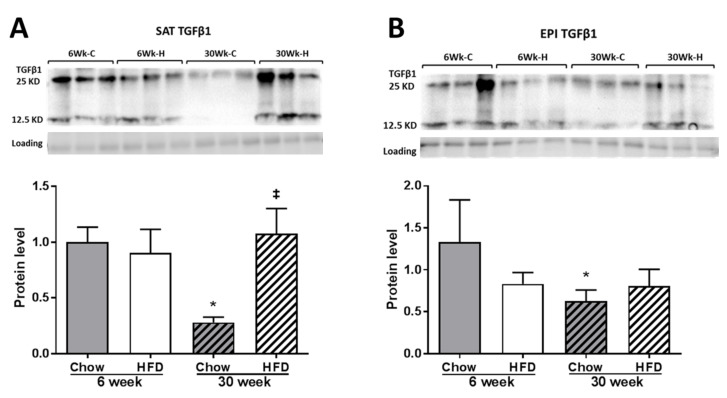
Western blot of TGFβ1 in SAT (**A**) and EPI (**B**). Data expressed as mean ± SD relative to 6 weeks Chow. Two-way ANOVA with Sidak’s multiple comparison test was used. *p*-value < 0.05 * vs. 6 weeks Chow, ‡ vs. 30 weeks Chow. TGFβ1 quantified with sum of two bands (12.5 and 25 kD).

We detected both the monomeric (12.5 KD) bioactive TGFβ1 protein and the dimeric form (25 KD) on Western immunoblot (Figure 10A,B). TGFβ1 protein levels remained comparable to Chow at week 6, but with increased exposure to HFD, an increase was observed in SAT but not in EPI.

### 2.5. Inflammatory Markers

**Figure 11:**
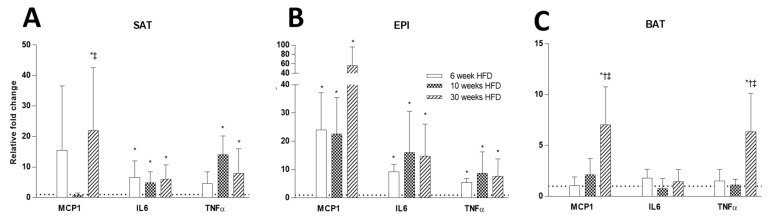
Comparison of mRNA levels of inflammatory markers (*Mcp1*, *Il6*, and *Tnfa*) at 6, 10, and 30 weeks HFD feeding in SAT (**A**), EPI (**B**), and BAT (**C**). Data expressed as mean ± SD relative to the respective Chow at each time point. An unpaired *t*-test was used to compare between Chow and HFD at each time point, and one-way ANOVA with Tukey’s multiple comparison test was used to compare among the HFD cohort of three different time points. *p*-value < 0.05 * vs. respective Chow, † vs. HFD 6 weeks, ‡ vs. HFD 10 weeks.

**Figure 12:**
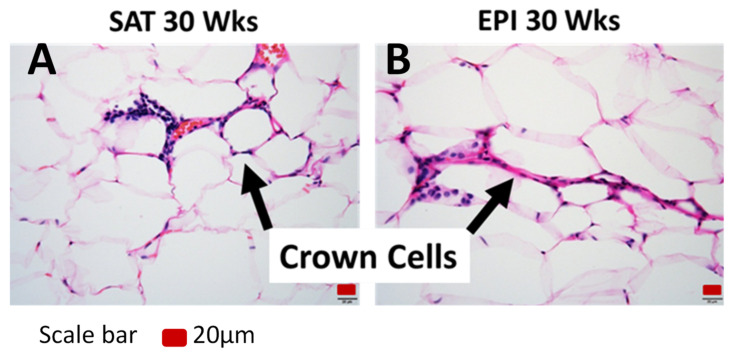
H&E staining to show crown cells in SAT (**A**) and EPI (**B**) at 30 weeks of HFD.

**Figure 13:**
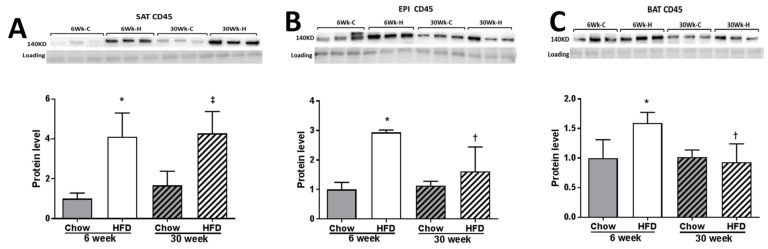
Western blot of CD45 in SAT (**A**), EPI (**B**), and BAT (**C**). Data expressed as mean ± SD relative to 6 weeks Chow. Two-way ANOVA with Sidak’s multiple comparison test was used. *p*-value < 0.05 * vs. respective Chow, † vs. HFD 6 weeks, ‡ vs. 30 weeks Chow.

The effect of HFD on inflammatory markers was far more pronounced in EPI than in SAT, increasing with the duration of HFD feeding. The effect of HFD on inflammatory markers was minimal in BAT. *Tnfa*, *Il6* and *Mcp1* mRNA were significantly increased in SAT and EPI over time with HFD (Figure 11A,B). A prolonged 30 weeks of HFD increased the numbers of “crownlike cells” in SAT and EPI, indicating the presence of dysfunctional adipocytes that were absent earlier (Figure 12A,B). Increased CD45 protein was consistently observed over the time course of the HFD in SAT (Figure 13A), but was only seen in EPI at week 6 (Figure 13B). However, it was not seen in EPI at week 30, which seemed inconsistent with the presence of crownlike cells at week 30. In BAT, CD45 protein was increased at week 6 HFD, but had decreased by week 30 (Figure 13C).

### 2.6. Tissue Insulin Resistance Markers

**Figure 14:**
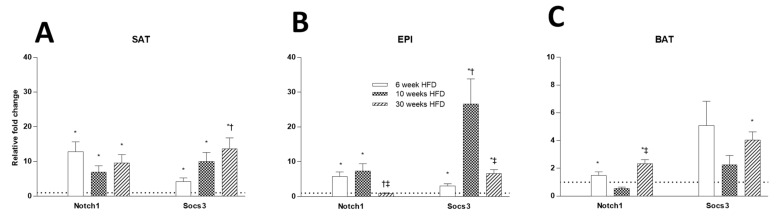
Comparison of tissue insulin resistance markers (*Notch1*, *Socs3*) at different time points in various fat depots at 6, 10, and 30 weeks of HFD feeding in SAT (**A**), EPI (**B**), and BAT (**C**). Data expressed as mean ± SD relative to the respective Chow at each time point. AN unpaired *t*-test was used to compare between Chow and HFD at each time point, and one-way ANOVA with Tukey’s multiple comparison test was used to compare among the HFD cohort of three different time points. *p*-value < 0.05 * vs. respective Chow, † vs. HFD 6 weeks, ‡ vs. HFD 10 weeks.

Consistent with increased insulin levels seen at all time points in HFD animals, tissue insulin resistance markers were also increased, but the patterns over time were different in SAT, EPI, and BAT (Figure 14A–C). In SAT, the mRNA levels of *Socs3* and *Notch1* were increased at all time points with an HFD. In EPI, a similar pattern was seen for Socs3, but not *Notch1*, where it was increased at 6 and 10 weeks, then had decreased by week 30. In BAT, the mRNA levels of *Socs3* and *Notch1* were only elevated at 30 weeks. Interestingly, the elevation of *Socs3* in SAT and BAT at 30 weeks was consistent with the changes in whole-body insulin resistance seen at this time of prolonged HFD feeding.

## 3. Discussion

This cross-sectional study at different time points of HFD feeding demonstrated a progression from normal functionality of all three fat depots through to dysfunctional fat tissue and the consequent increase in ectopic lipid in liver, and whole-body insulin resistance, seen as an increase in the area under the curve for the ITT, contributing to reduced ability to control blood glucose. The substantial length of this study design enabled us to understand how excess fat was initially handled by different adipose tissue depots and how their progressive dysfunctionality paralleled changes at a tissue and a whole-body level. The effect of the HFD on body weight, fat pad mass, liver weight, liver lipid content, and insulin resistance in our study occurred later than in other studies in which HFD feeding impaired insulin sensitivity earlier [20]. Over the course of our HFD study, there were distinct phases for the increases in body weight, fat pad mass, liver weight, and liver lipid content. Increased fat pad mass occurred early at 6 weeks in SAT, but was more sustained later in EPI. Overall, in EPI the mass of fat depots was greater in HFD, but all fat pads increased in both HFD and Chow. Increased lipid storage in SAT at 6 weeks reflected the expected ability to cope with excess calories. The major increase in liver weight and lipid content was seen after the fat cells had become dysfunctional (indicated by the presence of crown cells) at week 20 and 30 when the SAT, EPI, and BAT mass had plateaued. The time-dependent and tissue-preferential redistribution of lipids into different body tissues and organs observed in this study strongly supported the lipid spillover hypothesis [14,21].

The hypertrophic changes in SAT and EPI occurred early, at 6 weeks, and similar to another study [22], no further changes in size were seen from here to the end of the study at 30 weeks of HFD. Hypertrophic changes in adipose tissue were usually associated with the development of whole-body insulin resistance [23], which we observed later than has been seen in other studies [8]. The shift in the storage of lipids in adipose tissue to storage in ectopic tissues was dependent on the duration of HFD and occurred with the onset of systemic insulin resistance shown by the increase in the area under the ITT, the expression of adipose tissue insulin resistance markers (Socs3 and Notch1 mRNA), the inability of fat depots to increase in size, and the presence of crown cells, which indicated dysfunctionality in SAT and EPI depots.

At 6, 10, and 20 weeks of HFD, there were consistent increases in fat mass, but after 6 weeks, no further changes due to hypertrophy were seen; however, early on there were marked increases in the expression of genes regulating adipogenesis (i.e., *Pparg*, *Tle3*, adiponectin, and leptin) in SAT and EPI. With prolonged HFD feeding, the expression of genes regulating adipogenesis remained higher than Chow, but they decreased progressively over time. The highly upregulated Pref1 marker for preadipocytes in SAT would account for the relative abundance of preadipocytes observed, compared to EPI, and was consistent with the predicted greater adipogenic potential in SAT [24]. In BAT, the increased expression of genes regulating adipogenesis was seen only after 30 weeks of HFD, and coincided with appearance of multiple large lipid droplets in the tissue. The increased levels of adiponectin, leptin, resistin, *Tle3*, and *Pref1* in BAT could indicate either the recruitment of progenitor cells or that a change in fat cell phenotype had occurred, and signaled the appearance of increased white fat in BAT depots (best seen in the increases in WAT indicators leptin, resistin, and *Tle3*) (Figure 4C), an observation that had also been seen in other studies [25]. Furthermore, the increase in lipid accumulation in BAT and the exponential increase in liver lipid content corresponded with an inability to further increase the fat mass in SAT and EPI, suggesting that the function of BAT may have changed from its primary function of energy dissipation and heat production.

Earlier studies (Fromme et al.) had shown that the effect of HFD on thermogenic markers was highly variable [26], which we hypothesized to be due to differences in the fat depots examined, or to the shorter duration of HFD feeding. Our investigation revealed that there were novel depot-specific and time-course-related effects of the HFD. We showed that while mitochondrial biogenesis and thermogenic capacity markers were retained in both SAT and BAT at 6 weeks of HFD, less-pronounced effects were seen in EPI. Longer exposure to 30 weeks of HFD showed the thermogenic marker response of EPI remained constant from week 6; in SAT, it was reduced, but in BAT, it was increased. The increased thermogenic markers seen during early exposure to HFD in SAT and BAT would be consistent with a protective metabolic adaptation to burn excess calories. However, after a prolonged exposure to HFD, the decrease in thermogenic ability in SAT, in addition to EPI, may contribute to metabolic dysregulation and ectopic lipid deposition. We found the gene expression of *Ucp1* and *Prdm16* mRNA was increased over the duration of HFD, and this contrasted with the decreased protein levels. Such discordance between protein and mRNA findings has been reported in other studies due to numerous factors that included post-transcription translation efficiency or protein half-life, which had a poor correlation of 0.4 with these markers [27]. Further differences were seen in which higher *Pgc1a* mRNA was associated with increased *Ucp1* mRNA in SAT and BAT, but not in EPI. This indicated that in EPI, PGC1α may not regulate UCP1 protein expression in the same way as other depots. The mechanisms for these changes were not studied as part of this work, but require further investigation.

Like Fleury et al. [28], we demonstrated consistently higher *Ucp2* mRNA with HFD in SAT, EPI, and BAT. The thermogenic function of UCP2 has been highly contentious, but there was consensus that UCP2’s function was not primarily to promote thermogenesis [29]. Our observation of a progressive deterioration in metabolic function despite increased *Ucp2* suggested that UCP2 is unlikely to have the same metabolic protective or thermogenic function as UCP1. However, such a marked and progressive increase in *Ucp2* mRNA indicated that it may have a function other than energy regulation, such as the attenuation of free radical production by mitochondria to protect themselves from oxidative damage [29].

Studies have shown that collagen VI is highly upregulated in adipose tissue from obese individuals [16], and Choi et al. have shown increased collagen I, III, V, and VI mRNA by 16 weeks of HFD in EPI [10]. In our study, PSR staining indicated a greater fibrotic change in both SAT and EPI after prolonged exposure to HFD. Curiously, the increased gene expression of collagen VI and the profibrotic markers (*Tfgb1* and *Ccn2/Ctgf*) in SAT and EPI was greater at the earlier time points of HFD than it was in animals exposed to a longer HFD. While Western blots showed that fibrotic changes were detectable earlier in EPI at 6 weeks of HFD, the absence of apparent collagen accumulation by PSR in SAT at 6 weeks of HFD feeding, despite the increased profibrotic markers, suggested that there was increased ECM remodeling in SAT. This would be beneficial for the healthy expansion of SAT, as shown in other studies, where adipose tissue expansion was accompanied by increased ECM remodeling [30]. Further supporting this was our observation of increased *Timp1*, an inhibitor of ECM degradation [31], and decreased *Timp3*, an antifibrotic and anti-inflammatory marker [32], suggesting that this was not only due to the increased synthesis, but also decreased ECM degradation.

The remodeling of ECM observed in BAT was a novel aspect of our study. We observed only a minimal effect of HFD on BAT ECM remodeling during the earlier phase of feeding, but prolonged feeding revealed a significant upregulation of the genes for ECM remodeling (collagen VI, *Tgfb1*, *Ccn2/Ctgf*, *Timp1*, and *Timp3*). However, once again, there was discordance between gene expression and collagen accumulation in BAT, as evidenced by the unchanged PSR staining. Since increased potential for ECM remodeling without collagen accumulation coincided with the increased lipid accumulation in BAT, it again suggested that increased ECM remodeling in BAT may promote cellular expansion to increase lipid accumulation or promoted the increased presence of WAT in BAT, especially at the latter stages of HFD feeding. *Ccn2/Ctgf* increases were greater in SAT than in EPI, but the pattern was consistent with early increases followed by a decline at the latter weeks. In BAT, there was a sustained increase in *Ccn2/Ctgf*.

In our study, we found that marked fibrotic changes in SAT and EPI in the latter stages of HFD feeding coincided with the increased expression of inflammatory markers, increased macrophage infiltration and the formation of crownlike structures, supporting a role for inflammatory changes in triggering fibrotic changes [11]. Increased inflammatory markers observed in EPI were higher than in SAT, and would be consistent with a proinflammatory condition [33] and a higher risk of metabolic and cardiovascular disease seen in the visceral type of obesity [21]. However, inflammatory increases in *Tnfa* mRNA levels at an earlier phase of HFD feeding in SAT were not associated with significant ECM accumulation, but may be part of the essential role of an acute inflammatory response to allow adipose tissue remodeling and expansion to occur [13]. Keophiphath et al. also showed that proinflammatory microenvironments in human preadipocytes promoted increased proliferation of the cells [11], and Kimura et al. showed that the association with inflammation was greater in SAT than EPI [34]. As such, it would appear that the early inflammatory changes preceded and promoted healthy adipose tissue expansion in SAT. Higher levels of PRDM16 protein seen in EPI and SAT in chow-fed mice would be antagonistic to TGFβ1 and CCN2/CTGF’s profibrotic action, and may explain some of the variability in collagen deposition [35]. However, PRDM16 does maintain the progenitor population needed for tissue expansion [2].

This study found novel depot-specific and time-course-related effects of the HFD. Mitochondrial biogenesis and thermogenic capacity of SAT and BAT were retained in the early phases of HFD, but were not in EPI. Upon longer exposure to HFD, the thermogenic response in EPI did not change after week 6; in SAT, it reduced, but in BAT, it increased. This was also the first report of the effect of ECM remodeling in BAT. The HFD had a minimal effect on BAT ECM remodeling early, but ECM remodeling increased with a prolonged feeding, which coincided with the whitening of BAT. The extent of metabolic phenotyping across three fat depots and liver, along with development of insulin resistance, was comprehensive, which was in congruence with the lipid spillover hypothesis.

A limitation of this study was that intake was not measured. We used gene expression data and/or protein data to draw conclusions on adipogenesis, thermogenesis, insulin resistance, or whole-body metabolism generated without actual functional measurements on them. We did not use the gold standard for determining insulin resistance, the euglycemic hyperinsulinemic clamp, but the second most accepted method of assessing insulin resistance, the insulin tolerance test, which is simpler, less invasive, shorter (15 min), and uses a bolus of insulin instead of an infusion [36]. We also used epididymal fat pad in mice as a visceral fat. Other limitations of the study were that differences in the metabolism of mice and humans did not allow us to draw final comparisons to humans. In mice, the epididymal fat pads are derived from the same progenitor cells as most visceral adipose tissue [37], and have distinct metabolic and secretory factor differences from SAT and BAT. There is a very small depot of visceral adipose tissue in mice, and for this reason, EPI is usually studied in males. However, the similarity in the results from these studies to those done in humans was striking. We chose the C57BL6 mouse, as it is a well-known responder to the HFD, and has some shorter background studies that we could compare and contrast to this long time course of HFD study.

## 4. Materials and Methods

### 4.1. Animals and Diet

Male C57Bl/6J mice (*Mus musculus*) 4–5 weeks of age obtained from Animal Resource Centre, Western Australia, Perth were housed in Royal Prince Alfred Hospital animal house at the University of Sydney. The study was approved by the Animal Ethics Committee of the University of Sydney Animal Ethics Committee. Animals were quarantined for 1 week, randomized using a function in the Excel program (Microsoft), and grouped either as Chow or HFD, with 5 mice in the Chow groups and 7–8 in the HFD groups. Animals were either fed ad libitum on standard laboratory chow (12% fat) (meat-free mouse diet; Specialty Feeds^®^, Glen Forrest, WA, Australia) or the HFD (45% fat), which was prepared in-house with a formula based on rodent diet no. D12451 (Research Diets, New Brunswick, NJ, USA) (Table S1), as published by others [38,39]. Insulin sensitivity was determined using an insulin tolerance test (ITT) before the termination of animals as described previously [38].

Mice were euthanized with isoflurane at 6, 10, 20, and 30 weeks. The number of mice euthanized in the Chow control was 5 at all time points, but in HFD it was 7 mice at 6 and 10 weeks, and 8 mice at 20 and 30 weeks. SAT (taken from inguinal region), EPI (taken from epididymis), BAT (taken from intrascapular region), and liver were harvested from each animal. Blood was taken by cardiac puncture. Plasma was separated from blood cells and stored at −80 °C for the measurement of insulin. Snap-frozen adipose tissues and paraformaldehyde-fixed tissues were used for the analysis of tissue architecture. Plasma insulin was measured by an ELISA method (Merck Millipore, Darmstadt, Germany). Briefly, plasma (10 μL) was added to wells, followed by detection antibody (80 µL), and incubated at room temperature (RT) on a plate shaker (400 to 500 rpm) for 2 h. After washing with enzyme solution (streptavidin–horseradish peroxidase conjugate (100 µL) was added and incubated on a plate shaker at RT for 30 min. Again after washing, substrate solution (3, 3′, 5, 5′-tetramethylbenzidine; 100 µL) was added and incubated at RT on a plate shaker for 15 min. A stop solution (0.3 M HCl; 100 µL) was then added. The color developed was then read on a Tecan plate reader (Infinite M1000 PROTecan, Untersbergstr. 1A, Austria) at 450 nm and 590 nm. The absorbance units in each well was recorded, and plasma insulin concentration was determined by using a standard curve generated by plotting the absorbance of the insulin standards.

### 4.2. Measurement of Gene Expression

The gene expression for the markers of adipogenesis, thermogenesis, ECM remodeling, inflammation, and tissue insulin resistance were measured in each adipose tissue depot by qRT-PCR, as described previously [40,41]. Briefly, tissue was homogenized in a FastPrep Homogenizer (MP Biomedical, Irvine, CA, USA). RNA was extracted using an RNeasy Lipid Tissue Mini Kit (Qiagen, Hilden, Germany). Quantification and purity of RNA was measured by using the Nanodrop TM (Thermo-Fisher Scientific, Waltham, MA, USA), and RNA samples had a 260/280 ratio between 1.9–2.0. RNA (2 μg) was reverse transcribed using 50 pmol of oligo (dT)12–18 (Life Technologies, Carlsbad, CA, USA) and 0.4 pmol of random hexamers (Life Technologies). The resulting cDNA was aliquoted to 384-well plates using the Freedom EVO-2 100 (Tecan) automated platform. Following addition of Sensi MixTM SYBr^®^ (Bioline) and 500 nM of forward and reverse primers (Table 1), the samples were amplified on a Lightcycler 480 (Roche) programmed for 10 min at 95 °C, 40 cycles of 10 s at 95 °C, 15 s at 60 °C, 20 s at 72 °C, and then a hold at 4 °C. The mRNA levels of specific species were quantitated using the Delta/Delta method with NoNo used as the reference gene. The qRT-PCR results were expressed as fold change relative to their respective control.

### 4.3. Protein Quantification

Protein was extracted from 200 mg of snap-frozen adipose tissue (SAT and EPI) or 70 mg BAT for the quantification of UCP1 (catalog number ab10983, Abcam, Cambridge, UK), PRDM16 (catalog number ab106410, Abcam, Cambridge, UK), CD45 (catalog number ab10558, Abcam, Cambridge, UK), collagen VI (catalog number ab6588, Abcam, Cambridge, UK) and TGFβ1 (catalog number ab9758, Abcam, Cambridge, UK). The antibody dilution was 1:500 for all primary antibodies in all tissues, except for UCP1 in BAT (1:5000) and the secondary antibody (1:10,000; anti-rabbit IgG, catalog number S9169; Sigma^®^). As described earlier [40], tissues were homogenized in Eppendorf tubes containing 400 μL of RIPA buffer with a protease inhibitor cocktail (catalog number 04693159001 Roche, Rotkreuz, Switzerland) manually with a plastic pestle. Equal protein loading was made on gels, and band intensities were normalized to the total protein loaded, which was determined by the stain-free technique of Bio-Rad^®^.

**Table 1:** List of additional primers used for measurement of mRNA levels in adipose tissue in the study.

Genes	Forward	Reverse	Accession Number
*Pparg*	5′-CTGTCGGTTTCAGAAGTGCCT-3′	5′-CCCAAACCTGATGGCATTGTGAGACA-3′	NM_011671.4
*Tle3*	5′-TTGTCACAGGAGCATCAGCAG-3′	5′-CAGATTGGGGAGTCCACGTA-3′	NM_001083927.1
*Pref1*	5′-CCTGGCTGTGTCAATGGAGT-3′	5′-CTTGTGCTGGCAGTCCTTTC-3′	NM_001190705.1
Adiponectin	5′-CGACACCAAAAGGGCTCAGG-3′	5′-ACGTCATCTTCGGCATGACT-3′	NM_009605.4
Leptin	5′-GCTGCAAGGTGCAAGAAGAAG-3′	5′-TAGGACCAAAGCCACAGGAAC-3′	NM_008493.3
Resistin	5′-TTCCTGATGTCGGGGAAGTGA-3′	5′-GACCGGAGGACATCAGACATC-3′	NM_001204959.1
*Pgca1*	5′-CTGCGGGATGATGGAGACAG-3′	5′-TCGTTCGACCTGCGTAAAGT-3′	NM_008904.2
*Prdm16*	5′-TGACCATACCCGGAGGCATA-3′	5′-CTGACGAGGGTCCTGTGATG-3′	NM_001177995.1
*Tbx15*	5′-TGGCAGAAACAGAACTGGACT-3′	5′-CCTTGCTGCTTTTGCATGGT-3′	NM_009323.2
*Ucp1*	5′-CATGGGATCAAACCCCGCTA-3′	5′-ATTAGGGGTCGTCCCTTTCC-3′	NM_009463.3
*Ucp2*	5′-GGCCTCTGGAAAGGGACTTCT-3′	5′-TTGGCTTTCAGGAGAGTATCTTT-3′	NM_011671.4
*Mcp1*	5′-CACTCACCTGCTGCTACTCA-3′	5′-GCTTGGTGACAAAAACTACAGC-3′	NM_011333.3
*Il6*	5′-TCCTCTCTGCAAGAGACTTCC-3′	5′-TTGTGAAGTAGGGAAGGCCG-3′	NM_031168.1
*Tnfa*	5′-GACCCTCACACTCACAAACCA-3′	5′-ACAAGGTACAACCCATCGGC-3′	NM_001278601.1
Collagen VI	5′-GAACTTCCCTGCCAAACAGA-3′	5′-CACCTTGTGGAAGTTCTGCTC-3′	NM_146007.2
*Tgfb1*	5′-ACCGCAACAACGCCATCTAT-3′	5′-TGCTTCCCGAATGTCTGACG-3′	NM_011577.1
*Ccn2/ctgf*	5′-GAGTGTGCACTGCCAAAGATG-3′	5′-TCCAGGCAAGTGCATTGG T-3′	NM_010217.2
*Timp1*	5′-CACAAGTCCCAGAACCGC-3′	5′-GGATTCCGTGGCAGGC-3′	NM_001294280.2
*Timp3*	5′-CTTCTGCAACTCCGACATCGTGAT-3′	5′-CAGCAGGTACTGGTACTTGTTGAC-3′	NM_011595.2
*Notch1*	5′-ACAGTGCAACCCCCTGTATG-3′	5′-TCTAGGCCATCCCACTCACA-3′	NM_008714.3
*Socs3*	5′-TAGACTTCACGGCTGCCAAC-3′	5′-CGGGGAGCTAGTCCCGAA-3′	NM_007707.3
*Nono*	5′-TGCTCCTGTGCCACCTGGTACTC-3′	5′-CCGGAGCTGGACGGTTGAATGC-3′	NM_146007.2

### 4.4. Analysis of Tissue Structural Changes by Histochemistry

Adipose tissue fixed in 10% neutral buffered formalin was used for H and E staining, Picrosirius Red Staining (PSR) and immunohistochemical study as described previously [40]. H and E staining was used to measure the adipocyte size after imaging the entire hematoxylin stained section (n = 3/group), using an automated slide scanner (Olympus, Tokyo, Japan) and the imaging software, VS-DESKTOP Virtual Slide System (Olympus). PSR staining was used to detect collagen deposition in adipose tissue. Immunohistochemical staining for collagen VI (1:500; Catalog number ab6588, Abcam) was done after antigen retrieval by heating slides in a microwave oven. Secondary antibody (1:200, Biotinylated anti-rabbit IgG, catalog number BA-1000; Vector Laboratories^®^, Burlingame, CA, USA) was used followed by the addition of an avidin-biotin complex (Vectastain ABC kit, catalog number PK-4000; Vector Laboratories^®^) followed by 3, 3-Diaminobenzidine (DAB) (catalog number K3468; Dako^®^, Santa Clara, CA, USA).

### 4.5. Statistical Analysis

All data collected were entered into Prism GraphPad 7 software. We used an unpaired *t*-test, one-way ANOVA with Tukey’s multiple comparison test, and two-way ANOVA with Sidak’s multiple comparison test for analysis. Data were mainly expressed as mean ± SD, with the symbols indicating the differences that had achieved statistical significance at a level of *p* < 0.05.

## 5. Conclusions

In summary, we have shown that different fat depots had distinct phases in their responses to HFD in a prolonged-duration, ad libitum preclinical model of male C57Bl/6J mice. SAT appeared to have greater adipogenic, thermogenic, and ECM remodeling capacity early, rendering a metabolically protective function compared with EPI. These may be adaptive changes by adipose tissues in order to accommodate the excess fat safely during early stages of HFD feeding. However, on prolonged HFD feeding, there was a significant decline in the adipogenic potential, in mitochondrial biogenesis, and in cellular thermogenesis. When accompanied by massive infiltration of macrophages, excessive ECM accumulation into the SAT and EPI, the presence of crown cells, and the absence of further increases in fat pad weights indicated adipose tissue expansion was restricted. The strong relationship between the onset of dysfunctionality in fat cells coincided with ectopic fat deposition and detectable insulin resistance; while not causative, it certainly was suggestive of a significant effect. Long-term effects of HFD included the increased presence of WAT in BAT depots, and may have reflected increased beiging, a change in phenotype, or a loss of the heat-dissipating function of the BAT tissue.

## Data Availability

Not applicable.

## Supplementary Materials

The following are available online at https://www.mdpi.com/article/10.3390/ijms222413639/s1.
