# Supplementary material for: The Effect of a Sustained High-Fat Diet on the Metabolism of White and Brown Adipose Tissue and Its Impact on Insulin Resistance: A Selected Time Point Cross-Sectional Study
Source: Int J Mol Sci. 2021 Dec 20;22(24):13639. doi: 10.3390/ijms222413639 (PMC8706763; doi:10.3390/ijms222413639)
Supplement: Supplementary file 1 [file ijms-22-13639-s001.zip › ijms-1475936-supplementary.pdf]

Table S1: Composition of the High Fat Diet

Content	Weight (gm)	kCal	%kCal
Starch	214.8	759.8	13.86
Sucrose	224	910.13	16.60
Bran	57	230.16	4.20
Casein	261	955.55	17.43
Gelatin	23	79.83	1.46
Lard	250	2210.8	40.32
Safflower oil	34	284.42	5.19
Cholesterol	6	0	0
Mineral mix	51	0	0
Methionine	3.4	13.6	0.25
Choline bitartrate	4.6	18.4	0.34
AIN vitamins	14.8	0	0
TOTAL	1168.2	5483.78	100.00

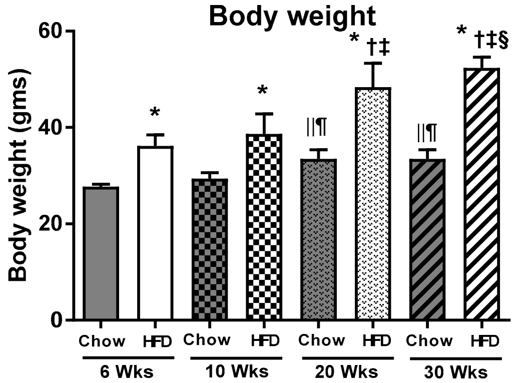

Figure S1: Body weight of chow and HFD at different time points. Data are expressed as Mean±SD. Unpaired t test was used to compare chow and HFD of each time point and One-way ANOVA with Tukey's multiple comparison test was used to compare among HFD across different time points.

p-value <0.05 * vs respective Chow, ‖ vs 6 weeks chow ¶ vs 10 weeks chow, ^ chow 20 weeks, † vs HFD 6 weeks and ‡ vs HFD 10 week § vs HFD 20 weeks.
